# Supplementary material for: Sleep selectively stabilizes contextual aspects of negative memories
Source: Sci Rep. 2018 Dec 14;8:17861. doi: 10.1038/s41598-018-35999-9 (PMC6294767; doi:10.1038/s41598-018-35999-9)
Supplement: Supplementary file 1 — Supplementary Information [file 41598_2018_35999_MOESM1_ESM.pdf]

Sleep selectively stabilizes contextual aspects of negative memories

Roy Cox, Marthe LV van Bronkhorst, Mollie Bayda, Herron Gomillion, Eileen Cho,
Mittie E Parr, Olivia P Manickas-Hill, Anna C Schapiro, Robert Stickgold

Supplementary Item Recognition

Table S1 shows absolute recognition scores at immediate and delayed tests for hit rate (HR), false alarm rate (FAR), and discriminability (d').

		SLEEP		WAKE		group		valence		group*valence	
	test	negative	neutral	negative	neutral	F	P	F	P	F	P
HR	T1	77.3 ± 11.2	74.0 ± 12.4	79.8 ± 10.7	77.9 ± 11.8	1.4	0.243	**5.0**	**0.028**	0.4	0.540
	T2	59.0 ± 17.9	58.0 ± 18.3	59.0 ± 17.4	57.4 ± 18.4	-	-	-	-	-	-
FAR	T1	38.2 ± 17.8	25.0 ± 14.7	30.8 ± 18.0	23.9 ± 15.4	1.2	0.27	**47.8**	**$<10^{-8}$**	**5.0**	**0.029**
	T2	30.5 ± 18.3	23.9 ± 16.9	26.1 ± 20.0	22.4 ± 19.2	-	-	-	-	-	-
d'	T1	1.1 ± 0.4	1.5 ± 0.5	1.5 ± 0.6	1.6 ± 0.7	**5.4**	**0.02**	**18.0**	**$<10^{-4}$**	2.6	0.11
	T2	0.8 ± 0.5	1.1 ± 0.5	1.0 ± 0.7	1.2 ± 0.6	-	-	-	-	-	-

Table S1. Recognition performance (mean ± SD). T1: immediate test, T2: delayed test. F test degrees of freedom: (1,69). Significant effects indicated in bold.

Immediate Test

We assessed baseline recognition performance at immediate test as a function of GROUP (sleep/wake) and VALENCE (negative/neutral) for each recognition metric. See Table S1 for condition averages and ANOVA results.

HR was significantly greater than chance (50%) in each condition (one-sample t tests: all $P<10^{-10}$), indicating successful encoding. We observed a small but significant main effect of VALENCE, consistent with the typical benefit for emotional memories. While absolute differences were similar for both groups, post hoc tests indicated a significant effect for the sleep ($t(45)=2.2$, $P=0.03$), but not the wake group ($t(24)=1.2$, $P=0.23$). Importantly, we did not observe an effect of GROUP or GROUP*VALENCE interaction, indicating comparable encoding success across sleep and wake. When adding SIDE (negative-left/negative-right) as a between-subject factor, all effects involving this factor were non-significant (all $F(1,67)<0.5$, all $P>0.48$), indicating that the visual field where negative stimuli were presented at encoding did not impact subsequent recognition of old items.

FAR was significantly below chance (50%) in each condition (all $P<10^{-4}$), indicating successful rejection of novel items. Although FAR did not vary as a function of GROUP, it was significantly enhanced for negative items. Post hoc tests confirmed greater FAR for negative relative to neutral items in both groups (sleep: $t(45)=7.7$, $P<10^{-8}$; wake: $t(24)=2.9$, $P=0.007$). These results are consistent with previous observations that emotional information is more readily endorsed as old, resulting in increases in both FAR and HR[1]. Moreover, GROUP and VALENCE interacted such that the

FAR difference between negative and neutral items was greater in the sleep group. However, neither negative (t(69)=1.7, P=0.10) nor neutral items (t(69)=0.3, P=0.78) differed significantly between groups. Additional analyses involving SIDE did not yield significant effects (all F(1,67)<0.54, all P>0.46).

Discriminability scores (d') were significantly above zero in each condition (all P<10^{-11}). As a consequence of the enhanced FAR for negative items, subjects' ability to discriminate old from new items was significantly reduced for negative relative to neutral items (main effect of VALENCE), with post hoc tests reaching significance for the sleep (t(45)=5.1, P<10^{-5}) but not the wake (t(24)=1.6, P=0.13) group. The low d' for the negative-sleep condition drove a main effect of GROUP, which follow-up tests indicated to be significant for negative (t(69)=3.1, P=0.003) but not neutral items (t(69)=1.3, P=0.20). Despite this, no significant GROUP*VALENCE interaction emerged. Finally, adding SIDE as a factor did not result in significant effects (all F(1,67)<1.7, all P>0.20).

In sum, while there were no baseline group differences in HR, greater FAR in the negative-sleep condition resulted in higher initial d' scores in the wake group. However, as d' change scores across the 12 h interval did not differ significantly between groups, this (potentially circadian) effect does not pose a major concern.

Change over 12 h

As reported in the main article (section *Item Recognition – Change over 12 h*; Table 3), both HR and d' showed robust decreases across 12 h in each condition but were unaffected by GROUP or VALENCE. In contrast, FAR was relatively stable across this interval (Table S2), showing a significant reduction in the negative-sleep condition only (one-sample t tests vs. zero: t(45)=2.8, P=0.008; all other P>0.10). The ANOVA revealed a significant main effect of VALENCE, with a post hoc analysis indicating a significant difference between negative and neutral items in the sleep (t(45)=3.3, P=0.002) but not the wake (t(24)=1.0, P=0.33) group.

	SLEEP		WAKE		group		valence		group*valence	
	negative	neutral	negative	neutral	F	P	F	P	F	P
FAR	–7.7 ± 19.0	–1.1 ± 15.2	–4.7 ± 13.8	–1.6 ± 14.1	0.1	0.72	**7.4**	**0.008**	1.0	0.33

Table S2. Change in false alarm rate across 12 h (mean ± SD). F test degrees of freedom: (1,69). Significant effects indicated in bold.

As mentioned in the previous section (*Supplementary Item Recognition – Immediate Test*), we observed higher baseline FARs for negative than neutral items, with this difference most pronounced in the sleep group. Interestingly, baseline FAR and FAR changes across the 12 h interval were negatively correlated, such that individuals with more false alarms at immediate test tended to show the largest reduction in FAR (negative: R=–0.43, P=0.0002; neutral: R=– 0.29, P=0.01; sleep and wake groups pooled), suggesting that the passage of time tended to suppress and thereby normalize FAR. Importantly, we observed no significant GROUP or GROUP*VALENCE interaction, indicating that changes in FAR were similar for sleep and wake. Adding SIDE as a factor did not result in additional significant effects (all F(1,67)<0.76, all P>0.38).

Supplementary Contextual Memory

Table S3 shows absolute performance of contextual memory for hits at immediate and delayed tests.

	SLEEP		WAKE		group		valence		group*valence	
test	negative	neutral	negative	neutral	F	P	F	P	F	P
T1	73.9 ± 14.0	74.7 ± 16.9	75.6 ± 16.5	78.3 ± 15.5	0.6	0.43	0.8	0.38	0.2	0.64
T2	74.0 ± 19.5	66.9 ± 19.2	64.6 ± 25.9	72.8 ± 19.6	-	-	-	-	-	-

Table S3. Contextual memory for hits (mean ± SD). T1: immediate test, T2: delayed test. F test degrees of freedom: (1,69).

Immediate Test

As reported in the main article (section *Contextual Memory – Immediate Test*), and also shown in Table S3, baseline contextual memory for hits did not differ as a function of GROUP or VALENCE. Unexpectedly, adding SIDE as a factor resulted in significant VALENCE*SIDE (F(1,67)=4.8, P=0.03) and GROUP*VALENCE*SIDE (F(1,67)=4.3, P=0.04) interactions (other effects involving SIDE: F(1,67)<0.9, both P>0.45). This effect appeared to be driven by higher performance for neutral items in the wake group when they had previously been presented in the left VF. Indeed, post hoc tests indicated a significant negative-neutral difference in this condition (paired t(10)=3.8, P=0.004), but none of the other three (all P>0.39). Note that this effect is opposite to what would be expected if the right hemisphere preferentially processes negative information (e.g., [2]). Given that this "wake-negative right" group contained the smallest number of subjects (N=11), the most likely explanation for this observation is a spurious effect, although a true effect cannot be ruled out.

Change over 12 h

We assessed whether SIDE (negative-left/negative-right) had an effect on our primary findings of selective stabilization of negative contextual memories across an interval of sleep. This analysis revealed a significant GROUP*SIDE interaction (F(1,67)=4.4, P=0.04), with no other effects involving SIDE reaching significance (all F(1,67)<1.4, all P>0.24). Closer inspection revealed that this effect was primarily driven by reduced forgetting for neutral items in the "wake-negative left" condition compared to neutral forgetting in the "wake-negative right" condition (independent t(23)=2.2, P=0.03). However, a similarly sized sleep benefit for negative relative to neutral memories was seen in the "negative-left" and "negative-right" groups (7.4 ± 24.4% vs. 8.5 ± 20.3%; t(44)=0.2, P=0.87), suggesting that the selective consolidation of negative memories during sleep did not depend importantly on presentation side.

Supplementary References

1. Dougal, S. & Rotello, C. M. ‘Remembering’ emotional words is based on response bias, not recollection. *Psychon. Bull. Rev.* **14,** 423–9 (2007).
2. Sato, W. & Aoki, S. Right hemispheric dominance in processing of unconscious negative emotion. *Brain Cogn.* **62,** 261–266 (2006).
